# ROS generated from biotic stress: Effects on plants and alleviation by endophytic microbes

**DOI:** 10.3389/fpls.2022.1042936

**Published:** 2022-10-24

**Authors:** Pramod Kumar Sahu, K. Jayalakshmi, Jyotsana Tilgam, Amrita Gupta, Yalavarthi Nagaraju, Adarsh Kumar, Saima Hamid, Harsh Vardhan Singh, Tatiana Minkina, Vishnu D. Rajput, Mahendra Vikram Singh Rajawat

**Affiliations:** ^1^ Indian Council of Agricultural Research (ICAR)-National Bureau of Agriculturally Important Microorganisms, Uttar Pradesh, India; ^2^ Plant Pathology, Indian Council of Agricultural Research (ICAR)-Directorate of Onion Garlic Research, Maharashtra, India; ^3^ Amity Institute of Biotechnology, Amity University Uttar Pradesh, Lucknow, India; ^4^ University of Kashmir, Srinagar, India; ^5^ Academy of Biology and Biotechnology, Southern Federal University, Rostov-on-Don, Russia

**Keywords:** ROS - reactive oxygen species, ROS homeostasis, induced systemic response (ISR), biotic stress, endophytes

## Abstract

Aerobic living is thought to generate reactive oxygen species (ROS), which are an inevitable chemical component. They are produced exclusively in cellular compartments in aerobic metabolism involving significant energy transfer and are regarded as by-products. ROS have a significant role in plant response to pathogenic stress, but the pattern varies between necrotrophs and biotrophs. A fine-tuned systemic induction system is involved in ROS-mediated disease development in plants. In regulated concentrations, ROS act as a signaling molecule and activate different pathways to suppress the pathogens. However, an excess of these ROS is deleterious to the plant system. Along with altering cell structure, ROS cause a variety of physiological reactions in plants that lower plant yield. ROS also degrade proteins, enzymes, nucleic acids, and other substances. Plants have their own mechanisms to overcome excess ROS and maintain homeostasis. Microbes, especially endophytes, have been reported to maintain ROS homeostasis in both biotic and abiotic stresses by multiple mechanisms. Endophytes themselves produce antioxidant compounds and also induce host plant machinery to supplement ROS scavenging. The structured reviews on how endophytes play a role in ROS homeostasis under biotic stress were very meager, so an attempt was made to compile the recent developments in ROS homeostasis using endophytes. This review deals with ROS production, mechanisms involved in ROS signaling, host plant mechanisms in alleviating oxidative stress, and the roles of endophytes in maintaining ROS homeostasis under biotic stress.

## 1 Introduction

**Figure 1:**
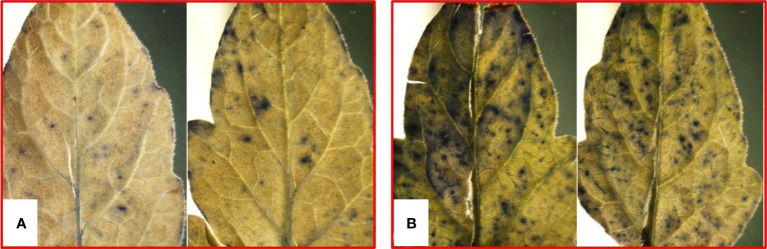
ROS accumulation in tomato leaves due to pathogenic stress as appeared from staining with nitroblue tetrazolium: **(A)** control and **(B)** pathogen inoculation.

Among the environmental stresses, biotic stress is a real scourge that causes enormous crop yield losses. Biotic factors, including microbial pathogens, weeds, and herbivores, restrict the plants’ ability to acquire their full genetic potential for vegetative and reproductive growth (Qaim, 2011; Ashraf et al., 2012). Being sessile, plants have evolved a surfeit of defense mechanisms that enable them to sense particular stresses and respond by triggering complex signaling networks that bring appropriate biochemical and physiological changes to surmount the stress (Atkinson and Urwin, 2012; Lamers et al., 2020). It is well established that reactive oxygen species (ROS) play an integral part in stress response and plant defense against pathogenic stress (Heller and Tudzynski, 2011; Srivastava et al., 2016; Sahu et al., 2020). Under normal growth conditions, plants use ROS in small concentrations as a signaling molecule (Baxter et al., 2014), but plants must maintain a perfect balance between ROS synthesis and ROS-scavenging mechanisms. Antioxidant enzymes are transcriptionally activated when plants are subjected to unfavorable environmental circumstances over an extended period. Rapid ROS synthesis necessitates activating the antioxidant defense system right away, which can be accomplished through retrograde signaling, redox-based changes, and activating ROS-scavenging enzymes. The main effect of ROS buildup is its ability to oxidize proteins that might serve as signaling targets, including kinases, transcription factors (TFs), and proteins involved in the stress response. Because ROS may alter the redox state of proteins by oxidizing methionine residues and cysteine thiol groups, they have the power to influence signaling. This causes ROS targets to become or become less functional, change in structure, and become or become less activated (Waszczak et al., 2015). Once ROS levels have significantly increased, they may have negative oxidative effects on proteins, nucleic acids, and lipids that finally lead to cell death (**Figure 1**). ROS is a component of tightly controlled programmed cell death (Petrov et al., 2015).

The perception of pathogen-associated molecular patterns (PAMPs) by pattern recognition receptors of plants causes the two-tiered plant immune system to react spontaneously, which activates the first line of defenses, including the oxidative burst, callose deposition, and enhanced expression of the PR gene, collectively known as PAMP-triggered immunity. A typical oxidative burst is the result of the activation of certain ROS-producing enzymes, including the nicotinamide adenine dinucleotide phosphat (NADPH) oxidases or cell wall peroxidases (PODs) (Mittler, 2002), causing the accumulation of an excessive amount of ROS. According to Zurbriggen et al. (2010), the oxidative burst in plants may cause the hypersensitive response (HR), which prevents the pathogen from spreading to nearby tissue. The HR can also act as a key signal to activate different pathways regulating plant defense responses and phytohormone synthesis, inhibiting further pathogen propagation and disease development (Beers and McDowell, 2001). It is widely known that ROS produced during plant responses to either abiotic stressors or pathogen infection stimulate mitogen-activated protein kinase (MAPK) signaling pathways. Furthermore, unrelated to the flagellin receptor FLAGELLIN SENSING 2, MPK7 downstream is activated by the ROS burst induced by pathogen invasion, which further activates genes linked to pathogenesis (Dóczi et al., 2007). In MAPK signaling cascades, which are composed of MAPKKKs (MAP3Ks), MAPKKs (MAP2Ks), and MAPKs that are sequentially phosphorylated, a variety of target proteins, including TFs, are either activated or inactivated (Liu and He, 2017). The high reduction state of these chemicals is associated with better plant resistance to severe stress conditions and antioxidant capability (Foyer and Noctor, 2011). The ability of ROS signaling to spread from cell to cell and transmit signals across great distances, also known as the ROS wave, is a crucial aspect of this transmission (Mittler et al., 2011). Because of systemic stress response, the locally produced signal is transferred to the regions that are not immediately affected by the stress. The ROS wave is implicated in the propagation of rapid systemic stress response along with other hormone- or electric signal–mediated signaling pathways (Gilroy et al., 2016; Fichman and Mittler, 2020).

Excessive ROS discharge in plants while interacting with a pathogen can strike both partners. However, plants protect themselves by maintaining a perfect balance between ROS synthesis and ROS-scavenging mechanisms. Plants accomplish the ROS equilibrium by enzymatic or non-enzymatic antioxidant defense mechanisms that tightly compartmentalize the regulation of ROS levels (Foyer and Noctor, 2020). Some metabolites have low molecular weights, e.g., glutathione, ascorbate, tocopherol, flavonoids, and carotenoids, and play a significant role in non-enzymatic antioxidant defense (Foyer and Noctor, 2020), firmly relating the cellular antioxidant capability with the preservation of redox equilibrium. Ascorbate and glutathione are necessary to maintain the ROS concentration at physiological levels and may directly break down ROS. In addition, they act as co-substrates for the ascorbate-glutathione cycle enzymes. Many antioxidant proteins are directly phosphorylated, primarily by post-translational modifications. Notably, phosphorylation occurs inside the ascorbate-glutathione cycle and regulates hydrogen peroxide (H_2_O_2_) breakdown. Several enzymatic antioxidants maintain ROS homeostasis in plant cells. Among the enzymatic systems, superoxide dismutase (SOD) is considered the first line of defense to counter ROS-induced oxidative damage in nearly all living cells. Catalase (CAT) is involved in several plant physiological responses during vegetative and reproductive stages (Yang et al., 2019; Zhang et al., 2020). Furthermore, glutathione PODs and thio-, gluta-, and peroxiredoxins are also potent ROS scavengers (Kang et al., 2019). The induced levels of protective ROS scavengers are responsible for the tolerance level against biotic and abiotic stress. Moreover, endophytic microbes that reside inside the plant host contribute to pathogen defense status by mediating and regulating cellular redox homeostasis (2020; Sahu et al., 2019; Singh et al., 2020). Endophytes reduce ROS content in plant cells by increasing scavenging *via* increased glutathione and ascorbate redox state and promoting antioxidant enzyme activities (Sadeghi et al., 2020). A plant growth-promoting endophyte, *Piriformospora indica*, was reported to induce resistance to fungal diseases in barley. *P. indica* caused the elevated antioxidative level by activating the glutathione-ascorbate cycle (Waller et al., 2005). The ROS-scavenging enzymes such as SOD, POD, and CAT were also found to be overaccumulating in the plant colonized by *P. indica* (Trivedi et al., 2016). Like *P. indica*, this review has attempted to compile some recent examples illustrating the role of endophytes in ROS homeostasis with highlights on ROS production, mechanisms involved in ROS signaling, and host plant mechanisms in alleviating oxidative stress.

## 2 Reactive oxygen species in response to biotic stress

**Figure 2:**
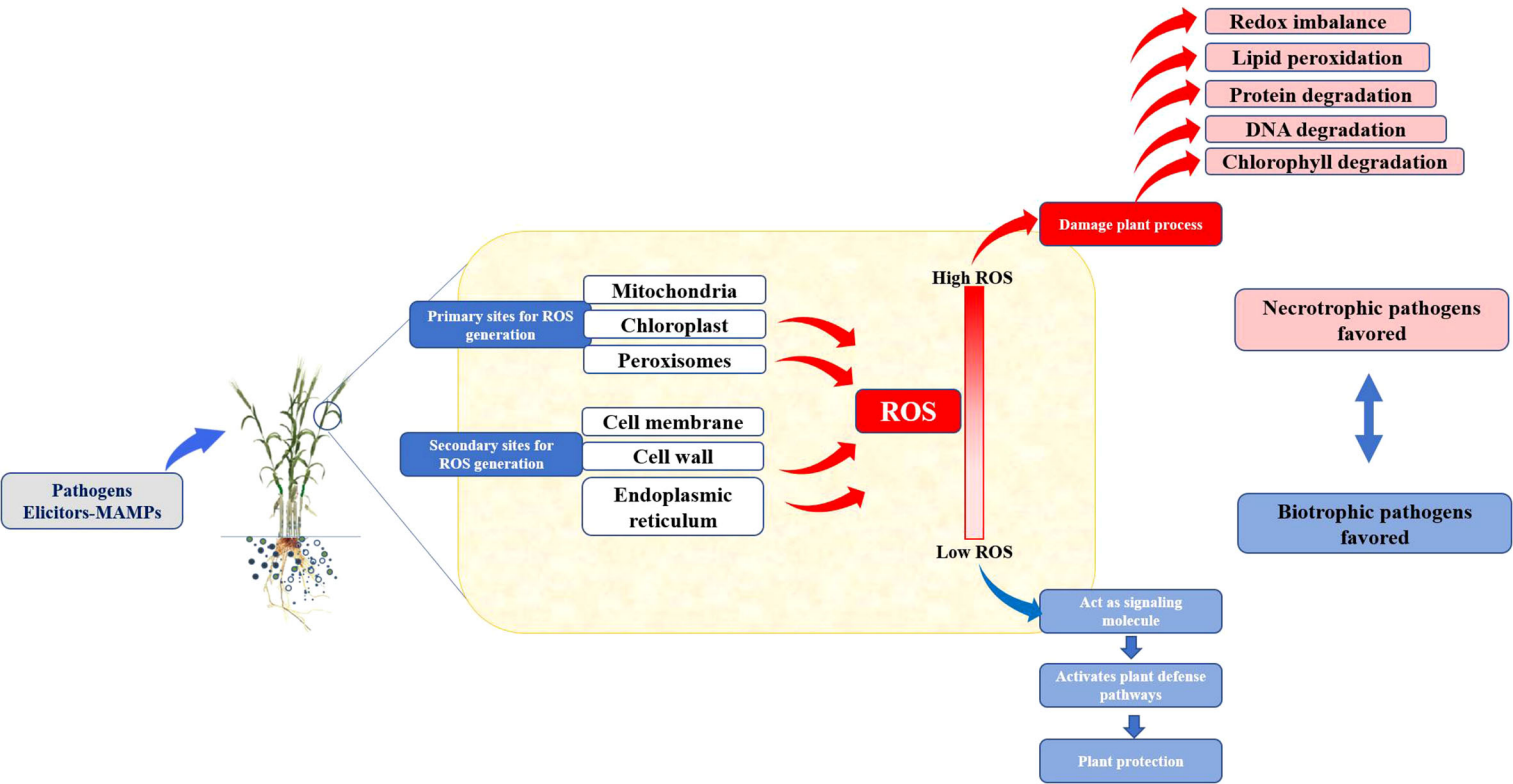
Response of biotrophic and nectrotropic pathogens to the ROS burst in host plants.

During crop growth, multiple biotic stresses such as pathogens and herbivores severely limit the crop productivity by altering various biochemical, physiological, and metabolic processes. Among them, the ROS production is a very crucial signaling response against pest and pathogen attack (**Table 1**). The ROS acts as an alarm for the plant’s metabolic pathways to divert toward protecting plant machinery and restricting damage. ROS is primarily produced in chloroplast, mitochondria, and peroxisomes (**Figure 2**). During stress, however, it is generated from secondary sites like the cell membrane, cell wall, endoplasmic reticulum, and apoplast. The signaling during the stress causes excessive generation of ROS, which, in turn, damages plant cells by causing redox imbalance, lipid peroxidation, degradation of chlorophyll, nucleic acids, and proteins. Harmful effects of excessive ROS are curbed by plants’ scavenging mechanisms.

**Table 1:** Mechanism of ROS generation and detoxification in different cellular compartment of plant cell.

Reactive oxygen species (ROS)	Source of ROS	ROS scavengers
**In chloroplast**		
Singlet oxygen, ^1^O_2_	PS II over excitation	α-Tocopherol, β-carotene, proline, plastoquinone, D1 protein, and flavonoids
Superoxide, O_2_•−	PS I Mehler reaction, photosynthesis ETC	Cu/Mn SOD, Fe SOD, and alternate oxidase
Hydrogen peroxide, H_2_O_2_	Dismutation of superoxide	APX, GPX, MDHAR, Prx, and TPX
Hydroxyl radicals, •OH	Fenton/Haber–Weiss reaction	Flavonoids and proline
Lipid radicals	Lipid peroxidation	α-Tocopherol, carotenoids, and flavonoid
**In mitochondria**		
Superoxide, O_2_•−	Complex I and II of ETC	Mn SOD
Hydrogen peroxide, H_2_O_2_	Dismutation of superoxide	APX, CAT, GPX, MDHAR, and Prx
Hydroxyl radicals, •OH	Fenton/Haber–Weiss reaction	Flavonoids and proline
**In peroxisomes**		
Hydrogen peroxide, H_2_O_2_	Photorespiration (glycolate oxidase), fatty acid β-oxidation (acyl CoA oxidase), dismutation of superoxide, and purine metabolism (urate oxidase)	CAT, APX, GPX, and MDHAR
Hydroxyl radicals, •OH	Fenton/Haber–Weiss reaction	Flavonoids
Superoxide, O2•−	Purine metabolism (xanthine oxidase) and peroxisomal membrane polypeptides (membrane monodehydroascorbate reductase)	Cu/Mn SOD and APX
**In cytosol**		
Superoxide, O_2_•−	Phytohorme biosynthesis (Aldehyde oxidase), Purine metabolism (Xanthine dehydrogenase)	Cu/Mn SOD
Hydrogen peroxide, H_2_O_2_	Dismutation of superoxide	APX, GPX, and TPX
Lipid radicals	Lipid peroxidation	GPX and Proline
^1^O_2_	–	Flavonoids and proline
**In apoplast/cell wall**		
Hydrogen peroxide, H_2_O_2_	Class III Peroxidases (PRXs), Dismutation of superoxide, Oxalate oxidase, Amine oxidase, Polyamine oxidase, Germins	APX, GPX, and CAT
Hydroxyl radicals, •OH	Fenton/Haber–Weiss reaction	Flavonoids and proline
**In plasma membrane**		
Superoxide, O_2_•−	NADPH oxidase (RBOH homolog)	SOD
Lipid radicals	Lipid peroxidation	α-Tocopherol
**In endoplasmic reticulum**		
Superoxide, O_2_•−	NADPH-dependent cytochrome 450 and cytochrome 540	-
Hydrogen peroxide, H_2_O_2_	Dismutation of superoxide	–
**In vacuole**		
Hydrogen peroxide, H_2_O_2_	–	APX and flavonoids
^1^O_2_	–	Flavonoids
Hydroxyl radicals, •OH	–	Flavonoids

APX, ascorbate peroxidase; MDHAR, monodehydroascorbate reductase; CAT, catalase; GPX, glutathione peroxidase; TPX, thiorexiredoxin; Prx, peroxiredoxin; RBOH, respiratory burst oxidase homolog; SOD, superoxide dismutase.

On the other hand, pathogen infections cause certain ROS-producing enzymes, including the NADPH oxidases or cell wall PODs, to be activated (Mittler, 2002). These enzymes produce an excessive amount of ROS, known as the oxidative burst  (Heller and Tudzynski, 2011). According to Zurbriggen et al. (2010), the oxidative burst in plants may cause the HR, which prevents the pathogen from spreading to nearby tissue. The HR can also act as a key signal to activate different pathways regulating plant defense responses and phytohormone synthesis (Beers and McDowell, 2001), inhibiting further pathogen propagation and disease development. The sequential induction of these pathways results in alteration in expression of genes related to the plant defense system, induces formation of phytoalexins, and causes callose deposition. This altogether results in a resistance response against a pathogen (Forman et al., 2010). Plants typically enhance their antioxidant capacity in response to abiotic stress, which aids them in reestablishing the cellular redox equilibrium (Hazen et al., 2003; Buchanan and Balmer, 2005).

### 2.1 ROS and pathogenesis by necrotrophs and biotrophs

The biotrophic pathogens obtain their energy from live cells. These can be found on or in living hosts and have intricate nutrient needs that they derive from their hosts. Whereas, the necrotrophs get their energy from dead cells. They quickly enter and kill plant tissue and then feed on the decomposing host remains in a saprotrophic manner. In cases of biotic stress (El-Zahaby et al., 2004), a significant difference between necrotrophic and biotrophic pathogens could be observed. The pathogen-induced oxidative burst typically encourages disease development in host–pathogen interactions in necrotroph. In *Arabidopsis*, increased ROS levels (produced by treatments with xanthine/xanthine oxidase or glucose/glucose oxidase) accelerated *Botrytis cinerea*’s necrotrophic development and necrosis (Govrin and Levine, 2000). The *Botrytis*-induced necroses and fungal development were constrained by the inhibition of ROS using antioxidants or diphenylene iodonium (NADPH oxidase inhibitor) (Govrin and Levine, 2000). Similarly, with two necrotrophic fungi, *Pyrenophora teres* and *Rhynchosporium secalis*, antioxidant pre-treatment of barley hindered disease development (Able, 2003). Interestingly, infection by the *B. cinerea* in tomato decreases the level of antioxidants at the cellular and subcellular levels (chloroplastic and mitochondrial), which causes ROS to be over-absorbed, and this suppression aids in the successful fungus colonization (Kuzniak and Sklodowska, 2004).

These results seem to revalidate Farkas’ initial discovery that abiotic stress and infections caused by necrotrophic bacteria share an identical mode of action (Farkas, 1978). The observation is that tissue necrosis brought on by necrotrophic pathogens or abiotic factors exhibits subcellular indications of apoptosis [also called as programmed cell death (PCD)], such as damaging the cellular components (Li and Dickman, 2004). Furthermore, plants were given the ability to tolerate necrosis brought on by necrotrophic pathogens, abiotic stress, or chemical treatments exacerbating oxidative stress (menadione and hydrogen peroxide) by transgenic expression antiapoptotic genes  (Li and Dickman, 2004; Xu et al., 2004).

In contrast to necrotrophs or abiotic stress, ROS and antioxidants appear to have a different function in the pathogenesis brought on by biotrophic infections. Initially, when resistant plants are invaded by the incompatible biotrophic pathogens, the host’s antioxidant defense is inhibited (Mittler et al., 1998; Vanacker et al., 1998). It was proposed that the plant’s intricate defense system includes suppressing the antioxidants. The increasing ROS concentration may activate cellular signals that cause resistance and the plant to become hypersensitive. Leaves of sensitive barley developed HR-type symptoms when external hydrogen peroxide was administered (Hafez and Király, 2003). It appears crucial to emphasize that, in resistant (incompatible) host–pathogen interactions, ROS may also suppress or kill the pathogens in addition to their effect on the host’s manifestation of necrotic symptoms. Early hydrogen peroxide administration following powdery mildew infection of barley (before establishment) was demonstrated to kill the fungus, causing no HR development (El-Zahaby et al., 2004). The HR was, however, enhanced in the resistant plant if ROS (hydrogen peroxide) was inoculated before the pathogen infection.

Application of ROS to the resistant barley leaves after 2 days of inoculation, although HR was developed but could not stop the pathogen to cause necrosis (Bendahmane et al., 1999). Similarly, it could also be seen in potato cultivars having “exceptional resistance” toward the infection of potato virus X. The virus is thought to be destroyed extremely quickly after infection in potatoes that exhibit great resistance, preventing the resistant host from mounting a hypersensitive reaction (Bendahmane et al., 1999). Antioxidants are fully active, and ROS are scavenged in interactions between suitable hosts and biotrophic pathogens (El-Zahaby et al., 1995; Mittler et al., 1998). Because an effective host resistance response may be inhibited due to insufficient ROS levels, the pathogen may be able to spread illness. Hence, it seems that ROS plays a different role in biotic stress caused by biotrophic pathogens than it does in abiotic stress or stress caused by necrotrophic pathogens.

The investigation of *rboh* mutants and antisense lines provided genetic evidence for functional roles of NADPH oxidase *Rboh* during oxidative burst (Simon-Plas et al., 2002; Yoshioka et al., 2003). Extracellular peroxide production is eliminated when Rboh is downregulated or eliminated. However, HR and pathogen development are impacted differently by this lack of ROS. In response to avirulent bacteria, for instance, a reduced HR that was developed in double-mutant *Arabidopsis* (for *atrbohD* and *atrbohF* genes) exhibits lower HR (Torres et al., 2002). Similarly, *Nicotiana benthamiana* plants silenced by respiratory burst oxidase homologs (rboh genes: *NbrbohA* and *NbrbohB*) are less resistant to the avirulent *Phytophthora infestans*, where the reduction in HR was reported (Yoshioka et al., 2003). In contrary, the *Arabidopsis* mutant (for *atrbohF* gene) exhibited higher HR and showed higher resilience to a mildly virulent *Hyaloperonospora parasitica* strain (Torres et al., 2002). In addition, there is proof that several *Rboh* proteins share functional properties. For instance, the double-mutant *atrboh*D *atrboh*F in *Arabidopsis* emphasizes some features of the single *atrboh*D and *atrboh*F mutants (Kwak et al., 2003).

**Figure 3:**
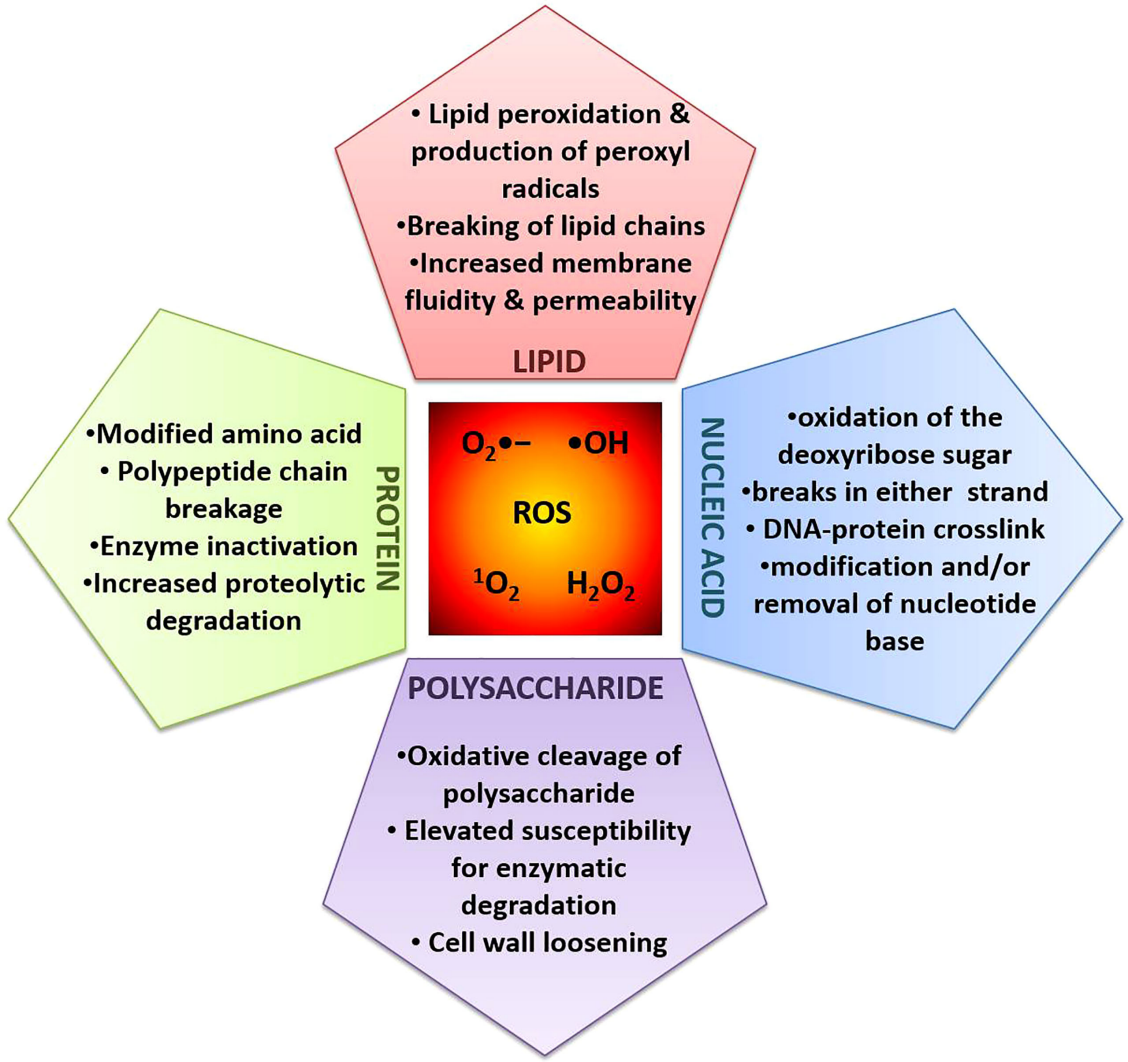
Biomolecular targets for ROS induced damage in plant system.

As a consequence of aerobic respiration, ROS are also produced by the pathogen itself (endogenous). They can also be found in the host environment (exogenous). In terms of eliminating pathogens, ROS have been referred to as “double-edged swords of life” (Mittler, 2017). First, ROS are considered to be the tool employed by both antibiotics and the host immune system because they have the ability to directly damage DNA, lipids, and proteins. Controversies, nevertheless, cast doubt on the paradigm. Second, effective pathogens use ROS for self-adaptation. Phototrophs are essential for maintaining life on Earth because they transform solar light energy into metabolic energy. They must pay a price for this in the form of the possibility of oxidative damage brought on by the many ROS produced as undesirable by-products, including H_2_O_2_, singlet oxygen (^1^O_2_), superoxide radical (O_2_•), and hydroxyl radical (OH•) (**Figure 3**). The ROS are produced using only 1%–2% of the total O_2_ used (Bhattacharjee, 2005).

## 3 Reactive oxygen species functioning under pathogenic stress

### 3.1 Singlet oxygen (^1^O_2_) under pathogenic stress

The ^1^O_2_ production was connected to the HR where host–pathogen interaction occurs at a race level (Vera-Estrella et al., 1992). In cell suspension culture, it has been observed that the extract of diseased leaves exhibited HR induced activated oxygen species (AOS) production in a period of 2 min. *Pseudomonas syringae* pv. *syringae*–infected tobacco plants displayed an HR accompanied by elevated production of O_2_ along with lipid peroxidation (Adam et al., 1989). Using the Cyt c reduction assay, ^1^O_2_ was found in leaf discs undergoing HR in investigations on the interaction between tobacco and the tobacco mosaic virus (Doke and Ohashi, 1988). However, in uninfected or systemically infected leaf discs, minimal ^1^O_2_ was found. A good histochemical test of AOS generation is created by the reduction of nitroblue tetrazolium by AOS. This test revealed that the ^1^O_2_-producing activity seemed to take place at the locations where a necrotic lesion would later develop. Beltran-García et al. (2014) showed that the melanin made by the fungus *Mycosphaerella fijiensis* causes the black Sigatoka disease in bananas and generated abundant singlet molecular oxygen and may act as a “photoactivated toxin” that kills cells in infected leaves and causes damaging symptoms.

### 3.2 Superoxide radical $\left({\text{O}}_{2}^{-}•\right)$ under pathogenic stress

Superoxide radicals (O_2_) are a by-product of peroxisomes’ regular metabolism. When molecular oxygen is reduced by one electron, superoxide, an anion radical, is created. The partitioning of superoxide radicals is catalyzed by the family of enzymes known as SODs. It is an essential enzyme needed to keep the cells’ redox potential stable. It is essential for shielding healthy cells from the ROS that are created when many intracellular pathogen infections occur. SOD eliminates excess ${\text{O}}_{2}^{-}$ radicals from the body by oxidizing to H_2_O_2_ and O_2_.

Kim et al. (1999) demonstrated that *Pseudomonas syringae* pv. *syringae* B728a mutants induced disease symptoms on bean pods and leaves similarly to the wild-type strain, despite more susceptibility of the mutants to the ${\text{O}}_{2}^{-}$ -producing chemical paraquat. Smith et al. (1996) had cloned a *sod* gene of *Xanthomonas campestris* pv. *campestris* encoding the main Mn-dependent SOD activity. However, efforts to create a SOD-deficient mutant were fruitless. The *sod* gene was activated *in planta* within 3 h of inoculation by these scientists using transcriptional fusions, with similar kinetics under compatible and incompatible contacts (Hugouvieux et al., 1998). However, it is probable that this strain’s Cu-Zn SOD output is enough to shield *P. syringae* from the toxicity of superoxide in plant leaves. Apart from pathogenic *P. syringae*, Cu-Zn SODs are also present in a variety of other plant associated bacteria, suggesting that these enzymes are widely conserved (Finn et al., 2010).

Other SOD subtypes, however, have been demonstrated to play a role in some plant–pathogen interactions. Mn SOD activity has been demonstrated to be necessary for the soft-rot pathogen *Erwinia chrysanthemi* to successfully macerate *Saintpaulia ionantha* leaves, but intriguingly, the Mn SOD mutants also preserved the capacity for potato maceration (Santos et al., 2001). This study by Santos et al. (2001) also discovered that thylakoid Cu-Zn SOD activity reduction and the light-dependent production of ROS are linked to the decrease in photosynthetic capacity. The formation of the necrotic lesions that distinguish the *P. syringae*–caused bacterial speck illness appears to depend on this ROS production. In *P. aeruginosa* PA14, an opportunistic pathogen, Mahajan-Miklos et al. (1999) discovered a gene that is critical for the quick destruction of the nematode *Caenorhabditis elegans* and is also implicated for causing disease in *Arabidopsis*. Under aerobic conditions, the phenazine toxin pyocyanin that this gene makes causes superoxide and hydrogen peroxide to form.

*Saccharomyces cerevisiae* grows slowly and is more sensitive to substances that produce ROS and H_2_O_2_ when *SOD*1 is lost (Moradas-Ferreira and Costa, 2000). *Erwinia chrysanthemi* cells are exposed to an oxidative environment at the beginning of an infection and need active defense mechanisms against oxidative damage, including MnSOD. Under these conditions, the pathogen may require iron homeostasis and active oxygen species detoxification to survive and spread disease (Santos et al., 2001).

*SOD*1 dysfunction can also change the typical physiology of fungi. As an example, the ability to produce conidia and mycorrhize is reduced in *Oidiodendron maius* (ericoid mycorrhizal fungus) *SOD*1 mutant (Abba et al., 2009). Later on, extracellular SODs were identified in a number of diseases (Robinett et al., 2018). Extracellular SODs in fungi typically have a glycosyl phosphatidyl inositol (GPI) anchor attachment site at the C-terminus and an N-terminal secretion signal peptide. The mature GPI anchor facilitates covalent attachment of the protein to the cell membrane and/or cell wall (Robinett et al., 2018). Such extracellular SODs from fungi are released to protect the ROS generated from host defensive response, providing survival in an environment with a lot of ROS. A Cu-only SOD (*Sod*5) is encoded in the *Candida albicans* genome, which helps the pathogen tolerate ROS (Gleason et al., 2014). The host’s copper co-factor is quickly bound and sequestered by the *C. albicans Sod*5, which reduces copper toxicity for *C. albicans* (Li et al., 2015). Similar to this, the dimorphic fungus *Histoplasma capsulatum*’s extracellular SOD helps yeast cells to resist oxidative stress caused by the host (Youseff et al., 2012). A secreted extracellular Zn-only SOD helps *Puccinia striiformis*, the fungus that causes stripe rust on wheat, to better withstand oxidative stress when it interacts with the wheat host (Liu et al., 2016). When conidia or conidiogensis are produced, *Colletotrichum graminicola* exhibits increased expression of the Mn SOD–expressing *SOD*2 gene (Fang et al., 2002). The biological purpose of *Fusarium oxysporum* f. sp. *vasinfectum*’s extracellular SOD *FoSod*5 is related to disease development. A *FoSOD*5 mutant’s pathogenicity on cotton was dramatically reduced because upregulation of *FoSOD*5 in cotton (Wang et al., 2021). In addition, they demonstrated that extracellular SODs play a role in the onset of ROS tolerance before harmful chemicals even reach the fungus cell. These results show that extracellular SODs are involved in pathogenesis in a variety of hosts and provide tolerance to the oxidative stress brought on by the host’s own ROS.

### 3.3 Hydrogen peroxide (H_2_O_2_) under pathogenic stress

Among all the ROS, H_2_O_2_ is the most stable. It is thought that plant mitochondria, which function as “energy factories”, are a significant site of H_2_O_2_ generation connected to ongoing physiological processes under aerobic conditions (Rasmusson et al., 1998). Another important source of H_2_O_2_ synthesis comes from chloroplasts. In the absence of transition metal ions, it weakly reacts with most organic molecules and could easily reach to the place far from its production site by diffusion through cell membrane. A rising corpus of research is showing that H_2_O_2_ is crucial for plants’ defense mechanisms under biotic stress. H_2_O_2_ is well established for impeding the growth and survival of plant pathogens, which further limit infection transmission (Yergaliyev et al., 2016). *In vitro* spore germination of various dangerous fungi was found to be suppressed by H_2_O_2_ at micromolar concentrations (Averyanov et al., 2007). The development of *Pseudomonas syringae* pv. *tabaci* was also revealed to be highly sensitive to micromolar doses of H_2_O_2_ (Cheng et al., 2016). The bacterial phytopathogen *Xanthomonas campestris* pv. *phaseoli* (Xp) infects plants. During the infection of the bacteria like *Xanthomonas*, plant generates H_2_O_2_ for defensive signaling and for restricting bacterial growth (Kumar et al., 2011).

In a similar study of bacterial infection, *Botrytis cinerea*’s mycelium growth was reduced by 50% when exposed to H_2_O_2_ (50 mmol/L) (Małolepsza and Urbanek, 1999). These H_2_O_2_ values are similar to those found in various pathosystems during the oxidative burst (Levine et al., 1994). Unexpectedly, compared to the pathogen, plant cells are relatively resilient to H_2_O_2_ (Lu and Higgins, 1999). H_2_O_2_ is relatively stable in biological systems compared with its usual precursor superoxide ( ${\text{O}}_{2}^{-}$ ); hence, it can be used as a relatively controllable substrate and signaling molecule. In plants, redox homeostasis is maintained through the balance between production and scavenging. Furthermore, transgenic plants having high constitutively expressed endogenous levels of H_2_O_2_ have shown improved disease resistance. The tobacco plants under light produced higher H_2_O_2_ and showed increased tolerance to *P. syringae* (Cheng et al., 2016). Potato plants added with glucose oxidase gene, which, in turn, produces H_2_O_2_, are found to show resistance toward *Phytophthora infestans* and *Erwinia carotovora* infection (Chamnongpol et al., 1998). CAT applied exogenously could negate the impact. On the basis of these results, it seems likely that higher levels of H_2_O_2_ made transgenic plants more resistant to both bacterial and fungal diseases.

Early plant–pathogen interactions produce H_2_O_2_, which acts as signaling molecule and induce cell wall, strengthening processes such as lignification, cross-linking of cell walls, production of papillae, and other cell wall–strengthening polymers. It has been discovered that papillae that accumulate callose, proteins, and phenolic chemicals are a significant barrier to pathogen penetration during the interactions between cereal and powdery mildew pathogen. According to cytological investigations, H_2_O_2_ is directly linked to the development of functional papillae in cells that the fungus pathogen has not yet been able to enter (Hückelhoven et al., 1999). The role of H_2_O_2_ as a substrate in lignification has received extensive documentation. Numerous plant–pathogen interactions have shown that an increase in H_2_O_2_ and the activity of PODs that oxidize phenylpropanoid alcohols add to the lignification process (Sahu et al., 2019). Furthermore, the concurrent oxidative burst-derived H_2_O_2_ acts as a mediator of the POD-catalyzed strengthening of hydroxyproline- and proline-containing structural proteins of cell wall that were triggered in cell suspension cultures (Otte and Barz, 1996). After the elicitor applied, the process of cell wall strengthening moved quickly and was over in a short amount of time. The highest H_2_O_2_ concentration in tomato leaves treated with *Fusarium* toxin correlated with the highest POD activity measured with syringaldazine and with ferulic acid. This syringaldazine is thought to be a marker of the lignification process, whereas the ferulic acid is considered to link polysaccharides and lignin in the cell wall (Kuiniak et al., 1999). Such cell wall–strengthening processes are quickly induced near the pathogen intrusion sites and restrict the nutrients availability to the pathogens. This also inhibits the transport of pathogenic toxins to host cell walls, making it highly resistant to the degrading enzymes produced by the pathogens, and, in turn, limits the spread of infection before transcription-dependent defense mechanisms develop. According to Shetty et al. (2007), the substantial H_2_O_2_ accumulation following pathogen infection in wheat shows that the *Septoria tritici* infection has passed from the biotrophic phase to the necrotrophic phase. Radwan et al. (2010) found that the H_2_O_2_ and malondialdehyde (MDA) levels were higher in Bean Yellow Mosaic Virus–infected *Vicia faba* leaves than in non-infected leaves.

### 3.4 Hydroxyl radical (OH•) under pathogenic stress

The OH• radical is considered as highly potent but is transient ROS. There is rising suggestion that the hydroxyl radical is much more than simply a disruptive agent, similar to how H_2_O_2_ was once assumed to be just a damaging oxidative metabolism by-product but now known to have a key role in signaling (Barba-Espín et al., 2011; Vavilala et al., 2015). In addition, it aids in stomatal closure, reproduction, the immune system’s reaction, and the ability to adapt to various kinds of stresses. Moreover, it also takes part in host cell death and essential in degradation (recycling) of plant waste. The formation of the OH• radical inside the cell is promoted by membrane and cell wall SOD, NADPH oxidases, and PODs along with transition metal catalysts. The activity of OH• radicals is precisely controlled for delivering substrates to the radical, because OH• radicals are having high diffusability and shorter half-life.

*Xanthomonas campestris* pv. *phaseoli* (Xp) is killed by H_2_O_2_ in the Fenton reaction as a result of the production of the hydroxyl radical. Xp was protected against H_2_O_2_ toxicity by substances that absorbed hydroxyl radicals, but not from superoxide or organic peroxide toxicity. Iron augmentation enhanced H_2_O_2_ killing. However, iron chelator pre-treatment of Xp had not provided any protective effects against H_2_O_2_ but, in fact, increased the concentration of H_2_O_2_ (Vattanaviboon and Mongkolsu, 1998). Rastogi and Pospisil (2012) demonstrated that the OH• was the most harmful ROS produced against *P. infestans* necrotrophic phase. It is generally known that OH• causes cell damage by lipid peroxidation, protein degradation, and nucleic acid damage by oxidizing biomolecules.

According to Karin and Stefan (2002), the tiny non-enzymatic agent known as the •OH is thought to have a role in the degradation of wood by brown rot. Inoculation of biocontrol agent *Pseudomonas fluorescens* increased the •OH generation in interaction with *Antrodia vaillantii* as compared to the fungus alone. However, contact with *Bacillus subtilis* had no effect on the amount of •OH produced. In the Fe-polyphenol catalyst made from coffee grounds, Morikawa (2018) isolated polyphenols from coffee, including caffeic acid and chlorogenic acid, which are crucial in the production of hydroxyl radicals. Application of H_2_O_2_ along with caffeic acid and chlorogenic acid in soil was found to minimize *Ralstonia solanacearum*–caused soil-borne illness in tomato cv. Momotaro. *Phaeoacremonium minimum* and *Phaeomoniella chlamydospora*, which produce esca disease in grape vines, promote the formation of hydroxyl radicals and may add to the disease development.

## 4 Detrimental effect of reactive oxygen species on plant physiology

The ROS family performs dual functions in plant physiology depending on their concentration. ROS regulate normal plant growth and development processes and act as signaling molecules to acclimate to environmental stress at low to moderate concentrations. However, at higher concentrations, they cause a risk that leads to cell injury and directly initiates untimely PCD (Sharma et al., 2012). ROS hinder several physiological functions of the plant cells through the alteration of DNA or RNA, oxidation of polyunsaturated fatty acids (PUFAs; lipid peroxidations), oxidations of amino acids in proteins, and deactivation of several enzymes through oxidation of co-factors (Mhamdi and Van Breusegem, 2018). During aerobic metabolism, ROS is formed as a by-product. A particular plant cell has a redox regulatory network maintained through ROS that regulates almost all processes, including gene expression and translation, metabolism, and turnover. In plant cells, ROS is conditionally produced in different subcellular compartments and cellular environments. Cells exploit ROS for signaling and sensing roles according to their spatial and temporal pattern along with reaction specificity. Furthermore, when the ROS level exceeds the threshold under adverse conditions, cells undergo oxidative stress, resulting in oxidative modification and cell toxicity. Various locations in the cell engage to generate ROS under both normal and stressful conditions. Organelles like mitochondria, chloroplasts, and peroxisomes represent potential sites for intracellular ROS synthesis. Whereas, NADPH oxidases, PODs, amine oxidases, etc., situated in plasma membrane and cell walls, are responsible for extracellular/apoplastic ROS generation (Kadota et al., 2014; Kadota et al., 2015). During plant–pathogen interaction, cell wall–localized enzymes are the main source of apoplastic ROS production.

In homeostasis, antioxidant machineries of plants scavenge excess ROS produced during various metabolic processes. The disturbance of the delicate balance between ROS generation and scavenging leads to enhanced generation of ROS. Such perturbation is caused by environmental stress, which inflates the ROS level in plant cells and leads to oxidative stress. Under stressful conditions, plants activate certain oxidases and PODs to produce ROS (Doke, 1985; Bolwell et al., 1998; Bolwell et al., 2002). The rapid rise of ROS concentration is referred to as an “oxidative burst”. Owing to high chemical activity, ROS have the potential to damage macromolecules such as pigments, nucleic acids, lipids, carbohydrates, and proteins. Excess ROS deteriorate the cell organelles and membrane components by disturbing membrane integrity, protein cross-linking, protein synthesis, ion transport, enzyme activity, DNA damage, etc., and, at severe levels, eventually triggering physiological or programmed cell death. ROS accumulation in chloroplasts is detrimental to chloroplast development, disrupts photosynthetic electron transport, and prevents photosystem II (PSII) assembly and repair.

### 4.1 ROS induced damage at the biomolecular level

Enhanced ROS accumulation causes damage to critical biomolecules including nucleic acid, lipid, protein, and polysaccharide, which alter cell physiology, membrane properties, and signaling cascades, eventually leading to death of the cells (**Figure 3**). Many reports suggest that transition metals (Fe, Cu, Cd, Ni, Cr, Pb, V, and Hg) are potent oxidative agents of biomolecules. These metals are more reactive toward oxidative damage as they are involved in enormous ROS production in cells (Stohs and Bagchi, 1995; Hemnani and Parihar, 1998).

Stress causes the production of damaging by-products that are bad for plants. ROS are substances created due to the reduction of oxygen molecules in plant tissue. These species include superoxide radicals, hydrogen peroxide (H_2_O_2_), hydroxyl radicals (OH•), and singlet oxygen (^1^O_2_). These oxygen radicals harm and kill cells by affecting proteins and lipids. When given ideal development conditions, the ROS levels within organelles are minimal. However, during stress, these levels are increased due to changes in cellular water potential that impact cellular homeostasis (Hussain et al., 2018). The balance between ROS production and scavenging allows cells to maintain homeostasis, with growth circumstances, the intensity and duration of stress, and other factors influencing cellular equilibrium (Beckhauser et al., 2016). The creation of ROS and its scavenging function is somewhat antagonistic; an excess of this molecule is poisonous to cells, whereas acting as a signal transducer, it activates the plant’s defense. As a result of the first burst of ROS generation, downstream post-stress activities are activated, which mobilize defense mechanisms and promote stress management (Sewelam et al., 2016). To prevent plant stress damage, the ROS-scavenging pathway is essential. Enhancing crop tolerance to environmental circumstances can be accomplished by comprehending the mechanisms of ROS generation, signaling, and scavenging (Chen et al., 2017).

Electron leakage produces ROS in the cell during photosynthesis and respiration. Plants have a well-regulated antioxidative mechanism combining enzymatic and non-enzymatic components that can balance ROS creation and scavenging to limit the overproduction of ROS and oxidative stress and to prevent cellular damage (Duan et al., 2012). Peroxides (POX), CAT, and SOD are a few of the enzymatic antioxidant systems that control the equilibrium of ROS in living things. These enzymes have a role in the conversion of oxygen to hydrogen peroxide. Ascorbic acids (AAs), tocopherol, flavonoids, glutathione, carotenoids, lipids, and phenolic compounds are non-enzymatic components that effectively mitigate oxidative damage by reducing ROS activity or by collaborating with enzymatic components to achieve efficient antioxidant activity through the utilization of H2O2 (Yin et al., 2015). Later, how these antioxidant systems and their components are regulated and how they work will be discussed.

The significance of controlling oxidative stress and local and systemic ROS signaling function in addressing abiotic stress has been thoroughly researched in recent years (de Carvalho et al., 2013). Despite these investigations, the information on antioxidant activity in various abiotically challenged plants still varies greatly. We still do not fully understand how abiotic stress management works or its essential elements. Here, we summarize plants’ systems for managing oxidative stress and their function in abiotic stress response. An overview of ROS generation in plants and how the plant system maintains ROS homeostasis are given in this paper. To achieve equilibrium, the connection between ROS generation and scavenging inside plant organelles is also briefly described. The list of genes involved in ROS regulation under abiotic stress is the most crucial part of this review. We need this information to find the processes and genes that control oxidative stress in plants and to choose the most important targets for crop breeding and genetic engineering.

#### 4.1.1 Nucleic acid

Among nucleic acids, organellar DNA is more prone to oxidative damage because it does not have protective histone proteins, unlike nuclear DNA, and are situated in direct vicinity of ROS generation sites. Irrespective of the source of DNA, damage leads to mutation and further results in abnormalities in the resultant protein, which influences different facets of cell physiology (Das and Roychoudhury, 2014). Some damages/lesions in DNA are subjected to repair processes and can be eliminated. However, a few errors are not easy to repair and can have biological consequences. Such an irreversible reaction happens when hydroxyl ions create DNA–protein cross-links between DNA and associated proteins. Hydroxyl radicals are likely to damage both the nitrogenous base and the deoxyribose sugar backbone. ROS deplete the H-atom from C-4 position of sugar moiety, resulting in radical formation that initiates single-strand breaks (Hemnani and Parihar, 1998). The oxidative damage of nucleotide bases results in various modification in DNA bases. Oxidative damage occurs when the oxygen atom binds with the carbon atom in the DNA, forming peroxyl radicles. Numerous covalent DNA changes, including single-nucleobase lesions, strand breaks, inter- and intrastrand cross-links, and protein–DNA cross-links, can be brought about by ROS. Purine damage is most frequently caused by 7,8-dihydro-8-oxoguanine, also known as 8-oxoguanine or 8-oxoG, whereas pyrimidine damage is most frequently caused by the creation of thymine glycol (Slupphaug et al., 2003). These lesions do not deform the helix and are not bulky. The 8,5′-cyclopurine-2′-deoxynucleosides are an illustration of bulky modified bases.

#### 4.1.2 Protein

Oxidative damage can have a devastating influence on protein structure, activity, and physical properties. Proteins can be modified directly or indirectly as a result of the damage. Direct modification includes chemical modifications like carbonylation, glutathionylation, nitrosylation, and disulfide bond formation. Indirect modification takes place when a protein molecule interacts with products of lipid peroxidation. Increased ROS concentrations in cells result in modifications in amino acids, cleavage of peptide chain, excessive cross-linking, alteration in electrical charges, and increasing protein degradation vulnerability (Davies, 1987; Sharma et al., 2012). The amino acids varied in susceptibility to oxidative damage. Sulfur- and thiol-containing amino acids are particularly vulnerable to ROS attack, according to research (Stohs and Bagchi, 1995). Metals were found to reduce the thiol groups in protein chains. Both singlet oxygen and hydroxyl radicals are detrimental to the amino acids Cys and Met. Superoxide radicals oxidize iron-sulfur centers of enzymes and inactivate them permanently. It is suggested that oxidized proteins are effective substrates for ubiquitination-mediated proteosomal degradation (Das and Roychoudhury, 2014).

#### 4.1.3 Lipid

Disruption in redox equilibrium leads to oxidative stress in the cell, mainly by enhanced lipid peroxidation. The lipid peroxidation is considered as a hallmark of oxidative damage and stress-induced cell injury. Oxidative stress-induced lipid degradation has been studied in different plants (Sharma and Dubey, 2005; Han and Yang, 2009; Mishra et al., 2011; Taïbi et al., 2016). Lipid peroxidation causes massive cell membrane damage by affecting the composition, assembly, structure, and dynamics of lipid membranes. As extremely reactive compound, lipid-derived radicals can also promote the subsequent ROS production, which interacts with nucleic acids and proteins (Gaschler and Stockwell, 2017). In lipid peroxidation, PUFAs (linoleic, linolenic, arachidonic, and docosahexaenoic acids) present in the membrane phospholipid are most susceptible to oxidation. ROS chiefly attacks the phospholipid moiety in two places: C-atom double bonds and ester linkages of fatty acids and glycerol. Superoxide and hydroxyl radicals interact with PUFA, producing peroxide that propagates the chain reaction and generates several other reactive species. As a consequence of elevated lipid peroxidation, membrane fluidity, and permeability increase, membrane localized enzymes, ion channels, and receptors become non-functional (Sharma et al., 2012; Das and Roychoudhury, 2014). MDA, a peroxidation product lipid of biomembrane, is considered as oxidative damage indicator of biomaterials (Yamauchi et al., 2008; Chen et al., 2015).

#### 4.1.4 Polysaccharides

ROS attack also causes fragmentation of polysaccharides of the cell wall, thereby probably changing the structure and function of these molecules. ROS such as singlet oxygen, hydroxyl radicals, and superoxide involve in oxidative cleavage of polysaccharides (Duan and Kasper, 2011; Faure et al., 2013; Lin et al., 2020). Reports have suggested that, in polysaccharide catabolism, prior treatment with ROS makes the substrate more suitable for enzymatic cleavage (Metcalfe et al., 1990; Faure et al., 2013). H_2_O_2_ elevates the activities of cell wall–degrading enzymes to increase the depolymerization of polysaccharide. In addition, OH• oxidize cell wall polysaccharides, causing the wall to loosen (Airianah et al., 2016).

## 5 ROS scavengers in plants

**Figure 4:**
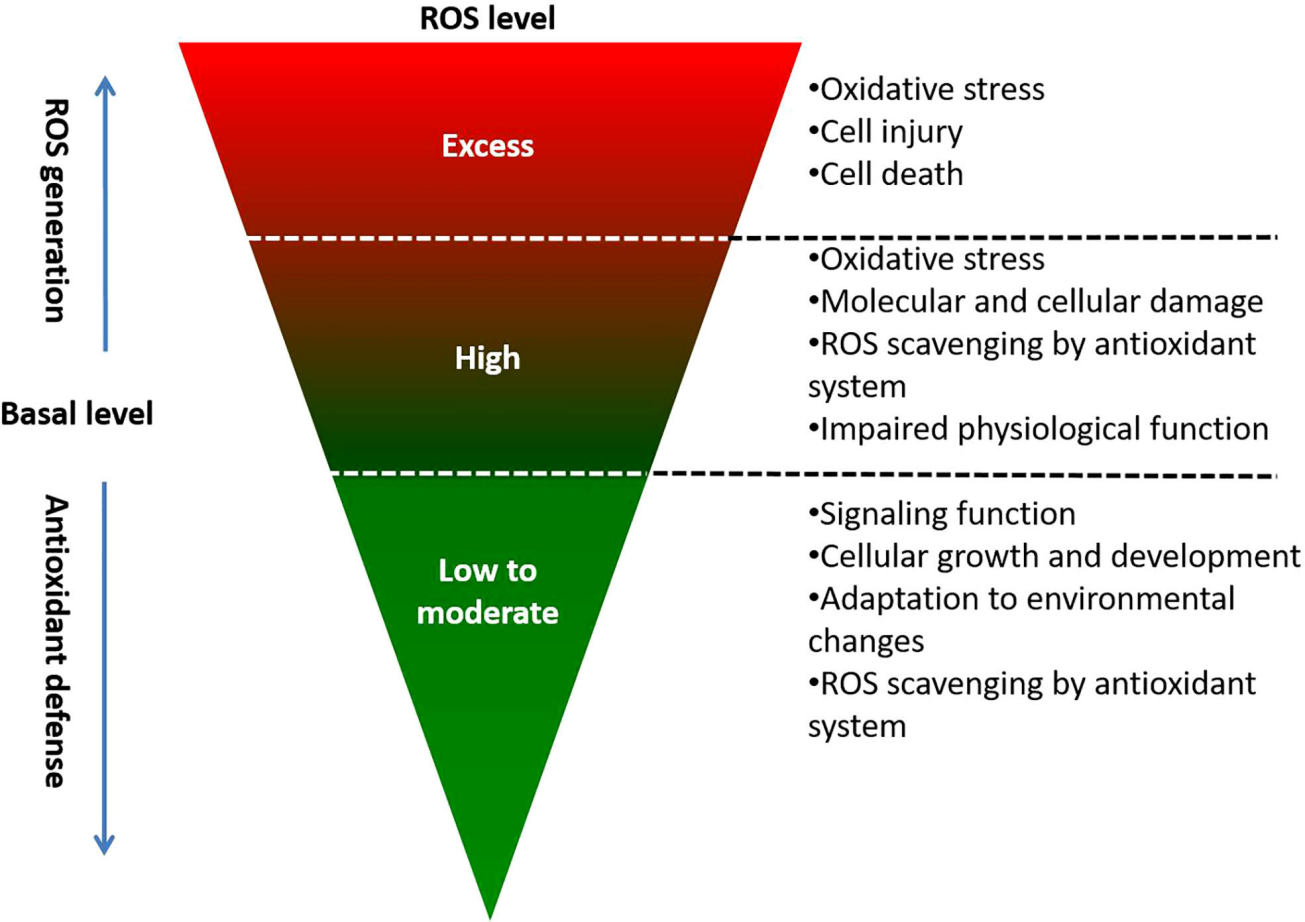
REDOX homeostasis and effect of various ROS levels on plant cellular function.

Plant systems possess their own antioxidant defense mechanism to detoxify overproduced ROS and protect the plant from oxidative stress under unfavorable development conditions. To maintain homeostasis in cells, enzymatic components and non-enzymatic antioxidants tightly control the ROS concentration and prevent any oxidative injury (**Figure 4**). Both mechanisms work synergistically and concurrently to counteract free radicals. Moreover, alternate oxidases present in ETC complex III of mitochondria control excessive ROS production indirectly (Rasmusson et al., 2009). Antioxidants perform a major function in the halting of oxidative chain reactions by degrading free radical intermediates.

### 5.1 Enzymatic antioxidants

Among the enzymatic systems, SOD is considered as the first line of defense to counter ROS-induced oxidative damage in nearly all living cells. It is a metalloenzyme, rapidly catalyzing the dismutation of superoxide radicals into dioxygen and hydrogen peroxide. In plants, three SOD isozymes have been reported on the basis of their metal co-factors: (i) Cu/Zn-SOD (found in cytosol, peroxisomes, and chloroplasts), (ii) Mn SOD (found in mitochondria), and (iii) Fe SOD (found in chloroplasts) (Sharma et al., 2012; Das and Roychoudhury, 2014). Overproduction of SOD is often correlated with enhanced oxidative stress tolerance in plants. Moreover, compartmentalization of different isozymes across the plant cell enables them to efficiently prevent stress. SOD is also capable of detoxifying the hydroxide radical to H_2_O_2_, and the generated H_2_O_2_ is subsequently degraded to water and dioxygen by CAT, ascorbate POD, and guaiacol POD (Anjum et al., 2016; Mittler, 2017).

The enzyme structure and metal co-factor binding allow for the classification of SOD enzymes into numerous types. The most prevalent SOD enzymes in eukaryotic cells are the cytosolic Cu/Zn SODs, and the fungal genome has at least one gene for Cu/Zn SOD synthesis (Miller, 2012). ROS can be used by pathogenic pseudomonads for their own purposes. For instance, several *P. syringae* pathovars produce the phytotoxin coronatine, which is known to be essential for the pathogen’s complete pathogenicity (Bender et al., 1987; Uppalapati et al., 2008).

CAT is an omnipresent heme enzyme, having more specificity for H_2_O_2_ but weaker specificity toward organic peroxides. CAT activity is prevalent in peroxisomes during different cellular processes and metabolizes H_2_O_2_ without requiring any cellular reducing equivalent. CAT enzyme has been found in chloroplasts, cytosols, and mitochondria, but its activity is less well understood. Similarly, guaiacol peroxidase (GPX), localized in the cytosol, vacuole, cell wall, and apoplast, is assumed to be an effective quencher of hydrogen peroxide under stress and non-stress conditions. It requires aromatic electron donors such as guaiacol and pyragallol (Asada 1992). GPX is involved in many indispensable activities, like lignin biosynthesis, ethylene biosynthesis, IAA degradation, wound repair, and protection against stress. Ascorbate peroxidase (APX) executes H_2_O_2_ scavenging functions in organelles such as chloroplasts, cytosol, mitochondria, and peroxisomes with higher affinity for H_2_O_2_. APX is a part of the ascorbate-glutathione cycle (that also maintains redox homeostasis in the plant) and harnesses ascorbate (AsA) as a specific electron donor for the reduction of H_2_O_2_ into water. In addition to APX, other enzymatic scavenger components include DHAR, MDHAR, glutathione reductase (GR), and nonenzymatic antioxidants such as AA and reduced glutathione (GSH) that play a role in the AsA-GSH cycle. MDHAR is a FAD enzyme that regenerates AA from the MDHA radical (formed in an APX-catalyzed reaction) by using NAD(P)H as a reducing substrate. MDHAR isozymes are produced in organelles like chloroplasts, cytosols, peroxisomes, mitochondria, and glyoxysomes. Another enzyme, DHAR, is responsible for regenerating the cellular AA pool by reducing dehydroascorbate (DHA) with the help of GSH as a reducing agent. DHAR enzyme is abundant in dry seeds, in roots, and in both etiolated and green shoots. GSH is oxidized to glutathione disulfide (GSSG) by the action of DHAR. GR is a NADPH-dependent enzyme that reduces GSSG to GSH, thereby keeping a higher cellular GSH to GSSG ratio.

### 5.2 Non-enzymatic antioxidants

Both ROS-scavenging systems function in a collaborative and interdependent way to increase cellular defense and to resist the oxidative damage resulting from ROS. Enzymatic antioxidants could not scavenge some highly reactive ROS such as O_2_ and OH·; therefore, plants depend on non-enzymatic antioxidants for scavenging such ROS. These non-enzymatic antioxidants comprise proline, phenolics, flavonoids, AA, GSH, alkaloids, α-tocopherol, carotenoids, etc. Non-enzymatic defense machineries also participate in normal plant physiological function by refining cellular processes like cell division, cell elongation, aging, and cell death, in addition to maintaining the cell redox homeostasis (Das and Roychoudhury, 2014). Non-enzymatic scavengers include relatively smaller organic molecules with low molecular weights. AA is found in abundance in most plant cell types, apoplasts, and organelles, particularly in photosynthetic tissues. It is known as a strong antioxidant as it can act as an electron donor and a reducing agent in different enzymatic and non-enzymatic reactions. Apoplastic AA is considered the first line of defense of the immune system against deleterious exogenous oxidants. AA offers membrane protection by immediate detoxification of hydrogen peroxide, peroxide radicals, hydroxyl radicals, and recycling of vitamin E (α-tocopherol) and carotenoids (Shao et al., 2008). AA also protects the biosynthetic enzymes from binding with prosthetic transition metal ions. When there is no stress, AA is present in the chloroplast in a reduced state where it helps in distributing excess excitation energy by functioning as a co-factor for enzyme violaxanthine de-epoxidase (Moussa et al., 2019). AA is oxidized in two successive processes: first turning into MDHA and then DHA. GSH was predominantly found in all cell components (Sharma et al., 2012).

Owing to its high reductive potential, GSH is involved in many biological activities such as cell growth/division, differentiation, aging, death, detoxification of xenobiotics, biosynthesis of nucleotides and proteins, enzymatic regulation, synthesis of phytochelatins for metal chelation, expression of stress-responsive genes, conjugation of metabolites, signal transduction, and regulation of sulphate transport. The equilibrium between the reduced glutathione and the GSSG is important for sustaining the redox state in cells. GSH performs its antioxidant role by different means. It detoxifies ROS members O_2_•−, •OH, H_2_O_2_ directly, prevent oxidative damage of macromolecules. GSH is involved in the recycling of antioxidant AsA by the AsA-GSH cycle and producing GSSG. The generated GSSG is converted back to GSH by employing NADPH as co-factor and an electron donor (Das and Roychoudhury, 2014). This process eventually restores the GSH pool in cells.

Vitamin E (α-tocopherol) is a member of the lipophilic antioxidant class, thus able to guard lipids containing biological membranes from oxidative damage. It safeguards the PSII structure and function by interacting with oxygen and quenching its surplus energy, thus protecting the lipid and other membrane components of the chloroplasts (Ivanov and Khorobrykh, 2003). The synthesis of vitamin E is restricted to photosynthetic organisms, including photosynthetic algae, and certain cyanobacteria. As an antioxidant, α-tocopherol effectively prevents the toxic effects of oxygen free radicals, singlet oxygen, and lipid peroxy radicals in cells (Moussa et al., 2019). Tocopherol functions as a potential trap for free radical by impeding the chain propagation during lipid autooxidation.

Carotenoids also belong to a class of lipophilic antioxidants that prevent oxidative damage caused by various ROS. Carotenoids exist not only in plants but also in microbes (Stahl and Sies, 2003). In plants, these come under the antennae molecule group absorbing 450- to 570-nm spectrum of the visible light and transfer excitation energy to chlorophyll. It is a highly potent physical and chemical quencher of singlet oxygen produced during photochemical reactions of photosynthesis and generates heat as a by-product (Krinsky, 1998; Shao et al., 2008). In addition, carotenoids interfere with radical-initiated reactions, especially with those that form peroxyl radicals. Thus, they protect the cellular membrane and lipoproteins of plant cells (Moussa et al., 2019). Carotenoid also plays a role as a precursor to signaling molecules that affect plant physiological function under stress and normal conditions. Similarly, flavonoid antioxidants are present in the plant kingdom as well as a few other photosynthetic organisms. It is categorized into four different classes based on their structure: flavonols, flavones, isoflavones, and anthocyanins. Anthocyanins are powerful inhibitors of lipid peroxidation. Flavonoids also avoid oxidative injury to nucleic acid brought by H_2_O_2_, HO., and ${\text{O}}_{2}^{-}$ It acts as a secondary ROS scavenger for photosynthetic tissues suffering damage because of excessive excitation energy. Flavonoids perform singlet oxygen quenching and mitigate the injury resulting in the chloroplast membrane (Agati et al., 2012). Flavonoid is an example of a phenolic compound, possessing ROS-scavenging property. In addition to flavonoids, other antioxidant phenolic compounds are tannins, hydroxycinnamate esters, and lignin. They show their scavenging role by trapping peroxyl radicals in lipid peroxidation and direct chelation with transition metal ions (Sharma et al., 2012). Another powerful antioxidant includes an osmolyte, i.e., proline. Proline is abundantly reported in different organisms to reduce the damaging effect of ROS under stress conditions (Das and Roychoudhury, 2014).

## 6 Role of endophytes in ROS homeostasis

Endophytes have been reported to maintain ROS homeostasis in both biotic (2020; Sahu et al., 2019; Singh et al., 2020) and abiotic stresses (Sahu et al., 2021). Endophytes decrease ROS content in the plant cells by increasing scavenging *via* increased redox state of glutathione and ascorbate along with promoting antioxidant enzyme activities (Sadeghi et al., 2020). Because endophytes have a close relationship with plants, they have a direct role in ROS homeostasis (**Table 2**). Reports suggested that the endophytes are present at the site of ROS production, i.e., plant cells (Thomas and Sekhar, 2014). The ROS helps establish mutualistic interaction between the endophyte, *Epichloe festucae*, and gross host, *Lolium perenne* (Tanaka et al., 2006). On the other hand, endophytic bacteria–produced ROS help to produce oxygenous sesquiterpenoids in *Atractylodes lancea.*

**Table 2:** Mechanism of ROS homeostasis by the endophytic microorganisms in plants.

SN.	Endophyte	Host	Biotic stress	Mechanism of ROS homeostasis	Reference
1.	*Acrophialophora jodhpurensis*	Tomato	Early blight	Antioxidant enzymes activity, phenolic content increased, and reduced cell death	Daroodi et al. (2021)
2.	*Piriformospora indica*	Bean plants	*Rhizoctonia solani*	Polyamines production	Kheyri et al. (2022)
3.	*Irpex lacteus*	*Distylium chinense* (Winter hazel)	*Penicillium*, *Candida* *albicans*, and *Aspergillus niger*	Antioxidant enzymes production	Duan et al. (2019)
4.	*B. amyloliquefaciens* and *P. fluorescens*	*W. somnifera*	*Alternaria alaternata*	Antioxidant machinery, such as SOD, CAT, PO, APx, GPx, and TFC	Mishra et al. (2018)
5.	AMF	Soybean	RKN–*Meloidogyne* genus	Glycosyltransferases, peroxidases, auxin-responsive proteins and gibberellin-regulated genes, and DELLA-like proteins	Beneventi et al. (2013)
6.	*Piriformispora indica*	Wheat, barely, and maize	*Fusarium verticillioides* and *Fusarium culmorum*	Antioxidants production	Kumar et al. (2009) and Waller et al. (2005)
7.	*Glomus mosseae*	*S. lycopersicum*	*Meloidogyne incognita*	ROS-scavenging mechanism	Voss et al. (2013)
8.	*Trichoderma harzianum*	*S. lycopersicum*	*Rhizoctonia solani*	Steroidal glycoalkaloids	Manganiello et al. (2018)
9.	*Pseudomonas aeruginosa*	Cucumber	*Sclerotium rolfsii*	Enhanced antioxidant activities and proline accumulation	Pandey et al. (2012)
10.	*Piriformospora indica*	Corn	*Fusarium* sp.	Improved antioxidant enzyme activity	Kumar et al. (2009)
11.	*Bacillus safensis*	Rice	*Rhizoctonia solani*	Antioxidant enzymes (SOD, POD, and APx) production	Sahu et al. (2020)
12.	*Bacillus* spp.	Tomato	*S. rolfsii*	PO, polyphenol oxidase (PPO), and APx reduce the accumulation of superoxides in tissues	Sahu et al. (2019)
13.	*B. amyloliquefaciens* NBRISN13		*Rhizoctonia solani*	ROS quenchers (arabitol, proline, mannitol, and phospholipases), production of non-metabolizable rare sugars (turanose) to keep the immune system induced and compromise fungal growth; activation of defense response through MAPK signaling and production of defense-related alkaloids such as terpenes and quinazoline, maintaining cell wall integrity, reduced starch-content, and the delayed formation of aerenchyma in parenchymal cells and delayed apoptosis	Srivastava et al. (2016)
14.	*Periconia* or *Microdochium*, *Microdochium*	Tallgrass prairie ecosystem		Polyphenol oxidases	Mandyam et al. (2010)
15.	*Phyllosticta* sp.	*Guazuma tomentosa*		Production of non-enzymatic antioxidants	Srinivasan et al. (2010)

Both pathogenic and helpful fungi encounter an oxidative surge of ROS during plant infection (Heller and Tudzynski, 2011). The ROS outburst results from plants’ innate immune response, which needs to be suppressed by the pathogen to prevent activating stronger plant defenses. Lack of an ROS production system can significantly affect the virulence and symbiosis of fungi with plants (Kayano et al., 2013). Fungi must combat both plant-derived ROS and ROS produced as a consequence of aerobic respiration. Strong antioxidant mechanism, including SOD, CAT, PO, GSH, and thioredoxin, has been created by fungal pathogens. Intriguingly, despite the fact that ROS was once assumed to function as a toxic agent to prevent pathogen infections, it is now well known that ROS signal the plant defensive response rather than being created in quantities high enough to defeat these pathogens (Marroquin-Guzman et al., 2017).

The focused production of endogenous ROS could be crucial for major phytopathogen developmental stages, and the absence of fungal ROS-producing systems may have an impact on virulence and plant–fungal symbioses (Kayano et al., 2013). Peroxiredoxins are frequently used by pathogens as a defense to reduce oxidative stress and maintain redox equilibrium (Stincone et al., 2014). Fungal phytopathogens also have other antioxidant systems such as CATs and glutathione PODs in addition to peroxiredoxins (Stincone et al., 2014; Breitenbach et al., 2015). Recently, it was found that oxidative stress induces the necrotrophic fungus *Ascochyta rabiei* to produce metabolic enzymes, potential effectors, and other virulence-related proteins (Maurya et al., 2020), suggesting a potential link between pathogenesis and stress response system, albeit it is yet unclear how fungal diseases penetrate the host plant despite the lethal oxidative burst. The only plausible conclusion is that fungal phytopathogens have developed innovative strategies to counteract the oxidative stress caused by the host during their evolution alongside it, and they have also taken advantage of this antagonism for their own gain.

On the other hand, *Nox*A (*Nox*1)– and *Nox*B (*Nox*2)–mediated fungal ROS production plays a crucial role in pathogen development and infection process. Plants have defense mechanisms against biotic and abiotic stresses, such as controlling the transit of ions like Na^+^ and K^+^, accumulating suitable solutes, and altering the expression of genes associated to stress. It has been proposed that the initial Ca^2+^ influx mediated by plasma membrane ion channels is essential for the adaptive signaling. It has also been proposed that ROS produced by NADPH oxidase (Nox) serve critical roles in controlling how various plant species, particularly halophytes, adapt to stress. One of the chief mechanisms used to counteract the plant defence system is NoxA and NoxB. These genes are involved in the production of H_2_O_2_ through the sequential reduction of oxygen. The ROS-producing Nox activity is demonstrated by respiratory burst oxidase homolog (Rboh) proteins, which are synergistically activated by Ca^2+^ binding to EF-hand motifs and Ca^2+^-dependent phosphorylation.

In some pathogens like *Magnaporthe oryzae*, oxidative burst by fungus is required to form the products related to the infection (Egan et al., 2007). In addition, the *Nox*1 and *Nox*2 complexes have a crucial role in determining the differences between different virulence determinants including appressoria and infection peg formation (Egan et al., 2007). However, the two homologs play different functions in plant infection.

ROS acts as signaling molecules for several environmental responses and conversely degenerate the cells, DNA, RNA, lipids, and protein. Hence, endophytic microorganisms and antioxidant compounds work together to help plants deal with stress (Mishra et al., 2014). Several endophytic microorganisms produce a myriad of compounds with antioxidant capacity. However, the information on the endophyte-produced antioxidant compounds was scattered and barely exposed. Hence, this section scouts the available literature for the ROS-scavenging compounds from endophytes.

The antioxidants are treasured for their critical role in plant disease management and ROS homeostasis. Plants and endophytes produce antioxidant compounds due to environmental stimuli, but the endophytes have supplementary action. Pioneering work on antioxidant compounds revealed that endophytic microbes produce phenols, flavonoids, tannins, hydroxyanthraquinones, phenolic terpenoids, etc. (White and Torres, 2010). Ethanolic extract of *Phyllosticta* sp. demonstrated strong antioxidant activity against both 2,2′-azino-bis-3-ethylbenzthiazoline-6-sulphonic acid and 1,1-diphenyl-2-picrylhydrazyl radicals (Srinivasan et al., 2010). Nevertheless, the endophyte-produced compounds play a significant role in antioxidant activity *in vitro* and *in vivo* because of their structural similarity (Kannan et al., 2016). Endophyte (*Pestalotiopsis microspore*)–derived antioxidants, pestatin (Harper et al., 2003) and isopestacin (Strobel, 2002), are potential scavengers of superoxide and hydroxyl radicles (Strobel and Daisy, 2003). The endophytic actinomycetes, *Streptomyces* sp. isolated from the *Mirabilis jalapa*, produce several antioxidant compounds (Passari et al., 2020).

Technological advancements in chromatography techniques are commonly employed for the analysis of crude extracts of endophyte (E+) plant extracts. These techniques helped to identify and characterize novel compounds with antioxidant properties. In Thin Layer Chromatography (TLC) and High Performance Liquid Chromatography (HPLC) analyses, several phenol-based antioxidants were classified as antioxidant compounds. Fungal endophytes were found to produce antioxidant compounds such as 2,2′-methylenebis, phenol, and some aldehydes, which could contribute to the host oxidative stress alleviation (Kaur et al., 2020).

## 7 Future prospects and conclusion in endophyte-mediated ROS homeostasis in plants

Endophytes are integral parts of ROS homeostasis in plants, and exploring some potential endophytes in the form of inoculants could be useful in reducing stress on the plants and achieving the production potential of crop plants. Endophytes with specific induction to an enzymatic or non-enzymatic antioxidant system must be identified through research. It would help in fitting such microbial inputs for precision agriculture in a calculated way.

Microbial inputs have wonderful roles in agriculture, and they are going to be multiplied many folds in the coming future. Endophytes are being characterized for their immense roles in growth promotion and stress tolerance. The induction of systemic resistance is one of the mechanisms by which the endophytes supplement biotic stress tolerance in the plants. When microbes inhabit host plants, they activate a number of pathways, which has an impact on plant metabolism. In such a scenario, it is indispensable to critically understand the mechanisms of microbe-mediated induction of plant pathways. This would facilitate the development of smart bio-formulations by reducing the energy-exhaustive induction of “not so important” pathways under specific pathogen stress. It could further be explored to design stress specific endophytic compounds to work against excess ROS and maintain normal plant growth and development. In other words, it would result in an energy-efficient microbial support system that could help plants sustain pathogenic stress conditions at the lowest energy cost.

## Author contributions

PS and MR contributed in conceptualization, methodology formulation and implementation, final draft preparation. JK, JT, AG, YN, AK, and SH contributed in survey of literature, graph preparation, and manuscript preparation. HS contributed in methodology and implementation. TM and VR contributed in editing of final draft of manuscript and language correction. All authors contributed to the article and approved the submitted version.

## Acknowledgments

The authors gratefully acknowledge their respective RPPs and Indian Council of Agricultural Research for providing the necessary facilities. VDR and TM acknowledge support by the laboratory «Soil Health» of the Southern Federal University with the financial support of the Ministry of Science and Higher Education of the Russian Federation, agreement No. 075-15-2022-1122.

## Conflict of interest

The authors declare that the research was conducted in the absence of any commercial or financial relationships that could be construed as a potential conflict of interest.

## Publisher’s note

All claims expressed in this article are solely those of the authors and do not necessarily represent those of their affiliated organizations, or those of the publisher, the editors and the reviewers. Any product that may be evaluated in this article, or claim that may be made by its manufacturer, is not guaranteed or endorsed by the publisher.
